# Supplementary material for: Short duration overnight cattle kraaling in natural rangelands: Does time after kraal use affect their utilization by wildlife and above ground grass parameters?
Source: PLoS One. 2022 Apr 28;17(4):e0248795. doi: 10.1371/journal.pone.0248795 (PMC9049567; doi:10.1371/journal.pone.0248795)
Supplement: Attachment — Submitted filename: response to reviewers.doc [file pone.0248795.s001.doc]

**Response to reviewer comments - PONE-D-21-06800**

Short duration overnight cattle kraaling in natural rangelands: does time after kraal use affect their utilization by wildlife and above ground grass parameters?

Dear Dr. Huruba

Thank you for submitting your manuscript to PLOS ONE. After careful consideration, we feel that it has merit but does not fully meet PLOS ONE’s publication criteria as it currently stands. Therefore, we invite you to submit a revised version of the manuscript that addresses the points raised during the review process.

The reviewers provide important feedback on this study, in particular, on clarifying the framing of the study, the details of the methodology used, and interpretation of results. I agree with the reviewers that the introduction needs some reorganization and streamlining, and that more detail is needed regarding the kraaling sites studied and the methods used in the study.

Wendy C. Turner
Academic Editor

3. Thank you for stating the following in the Financial Disclosure section:

'The authors received no specific funding for this work.'

We note that one or more of the authors are employed by a commercial company: E Oppenheimer & Son (Pty) Limited

a. Please provide an amended Funding Statement declaring this commercial affiliation, as well as a statement regarding the Role of Funders in your study. If the funding organization did not play a role in the study design, data collection and analysis, decision to publish, or preparation of the manuscript and only provided financial support in the form of authors' salaries and/or research materials, please review your statements relating to the author contributions, and ensure you have specifically and accurately indicated the role(s) that these authors had in your study. You can update author roles in the Author Contributions section of the online submission form.

Please also include the following statement within your amended Funding Statement.

“The funder provided support in the form of salaries for authors Rangarirai Huruba and Duncan N. MacFadyen, but did not have any additional role in the study design, data collection and analysis, decision to publish, or preparation of the manuscript. The specific roles of these authors are articulated in the ‘author contributions’ section.”

4. We note that Figure 1 in your submission contains map images which may be copyrighted – this image is ours.

All PLOS content is published under the Creative Commons Attribution License (CC BY 4.0), which means that the manuscript, images, and Supporting Information files will be freely available online, and any third party is permitted to access, download, copy, distribute, and use these materials in any way, even commercially, with proper attribution. For these reasons, we cannot publish previously copyrighted maps or satellite images created using proprietary data, such as Google software (Google Maps, Street View, and Earth). For more information, see our copyright guidelines: <http://journals.plos.org/plosone/s/licenses-and-copyright>.

 We require you to either (a) present written permission from the copyright holder to publish this figure specifically under the CC BY 4.0 license, or (b) remove the figure from your submission – this image has no copyright concerns. The authors are the copyright holders as they are the ones that produced it.

a. You may seek permission from the original copyright holder of Figure 1 to publish the content specifically under the CC BY 4.0 license. – this is not applicable.

We recommend that you contact the original copyright holder with the Content Permission Form (http://journals.plos.org/plosone/s/file?id=7c09/content-permission-form.pdf) and the following text:

“I request permission for the open-access journal PLOS ONE to publish XXX under the Creative Commons Attribution License (CCAL) CC BY 4.0 (http://creativecommons.org/licenses/by/4.0/). Please be aware that this license allows unrestricted use and distribution, even commercially, by third parties. Please reply and provide explicit written permission to publish XXX under a CC BY license and complete the attached form.”

Please upload the completed Content Permission Form or other proof of granted permissions as an "Other" file with your submission.

In the figure caption of the copyrighted figure, please include the following text: “Reprinted from [ref] under a CC BY license, with permission from [name of publisher], original copyright [original copyright year].”

b. If you are unable to obtain permission from the original copyright holder to publish this figure under the CC BY 4.0 license or if the copyright holder’s requirements are incompatible with the CC BY 4.0 license, please either i) remove the figure or ii) supply a replacement figure that complies with the CC BY 4.0 license. Please check copyright information on all replacement figures and update the figure caption with source information. If applicable, please specify in the figure caption text when a figure is similar but not identical to the original image and is therefore for illustrative purposes only.

The following resources for replacing copyrighted map figures may be helpful:

USGS National Map Viewer (public domain): http://viewer.nationalmap.gov/viewer/

The Gateway to Astronaut Photography of Earth (public domain): http://eol.jsc.nasa.gov/sseop/clickmap/

Maps at the CIA (public domain): https://www.cia.gov/library/publications/the-world-factbook/index.html and https://www.cia.gov/library/publications/cia-maps-publications/index.html

NASA Earth Observatory (public domain): http://earthobservatory.nasa.gov/

Landsat: <http://landsat.visibleearth.nasa.gov/>

USGS EROS (Earth Resources Observatory and Science (EROS) Center) (public domain): http://eros.usgs.gov/#

Natural Earth (public domain): http://www.naturalearthdata.com/

 5. Please include a copy of Table 3 which you refer to in your text on page 15. – it should be Table 1 & not table 3.

**Reviewers' comments:

Reviewer's Responses to Questions**

**Comments to the Author**

1. Is the manuscript technically sound, and do the data support the conclusions?

The manuscript must describe a technically sound piece of scientific research with data that supports the conclusions. Experiments must have been conducted rigorously, with appropriate controls, replication, and sample sizes. The conclusions must be drawn appropriately based on the data presented.

Reviewer #1: Partly

Reviewer #2: Partly

 2. Has the statistical analysis been performed appropriately and rigorously?

Reviewer #1: Yes

Reviewer #2: Yes

 3. Have the authors made all data underlying the findings in their manuscript fully available?

The [PLOS Data policy](http://www.plosone.org/static/policies.action#sharing) requires authors to make all data underlying the findings described in their manuscript fully available without restriction, with rare exception (please refer to the Data Availability Statement in the manuscript PDF file). The data should be provided as part of the manuscript or its supporting information, or deposited to a public repository. For example, in addition to summary statistics, the data points behind means, medians and variance measures should be available. If there are restrictions on publicly sharing data—e.g. participant privacy or use of data from a third party—those must be specified.

Reviewer #1: No

Reviewer #2: Yes

 4. Is the manuscript presented in an intelligible fashion and written in standard English?

PLOS ONE does not copyedit accepted manuscripts, so the language in submitted articles must be clear, correct, and unambiguous. Any typographical or grammatical errors should be corrected at revision, so please note any specific errors here.

 Reviewer #1: Yes

Reviewer #2: Yes

 5. Review Comments to the Author

Please use the space provided to explain your answers to the questions above. You may also include additional comments for the author, including concerns about dual publication, research ethics, or publication ethics. (Please upload your review as an attachment if it exceeds 20,000 characters)

**Reviewer #1**: I appreciate the opportunity to review this manuscript. The work examines wildlife visitation of abandoned cattle Kralls spanning from one- to four-year-old and also reports on above grass biomass accumulation in these heavily grazed sites. This work is of interest to a broad audience and underpins some of the benefits associated with both current and legacy effects of livestock wildlife integration in rangelands. The manuscript is fairly well written but may require some major revision before it gets to a level that is acceptable for publication. Particularly, I found the introduction not quite well done, and there are also some fundamental concerns on the design of the study. I have itemized some of these concerns below – the manuscript has been revised (see L16-257)

General Comments
First, the introduction section lacks of coherent flow between paragraphs. For example, I found it quite distracting switching from paragraph one (which talks about nutrient hotspots created by old cattle bomas) to paragraph 2 (which talks about effectiveness of camera trapping…. something that I actually don’t think should be presented here) to paragraph 3 (which revisits the same issues in paragraph 1) and later in paragraph 7 camera traps are revisited. Additionally, there is a lot of repetition and contradicting statements from one paragraph to another. I have pointed out some of these under specific comments below. Suffices to say, it is hard to read and follow though the introduction section as presented. – the introduction has been revised (see L67-257)

Secondly and most critical, there is little or no information relating to how the cattle bomas were set up. The authors present information about the age of the various bomas; which in real sense doesn’t tell much. For example, we know the trajectory of an abandoned boma depends on many factors including; i) the amount of time it was occupied, ii) what livestock species occupied it, iii) how many individual animals were present, iv) the prevailing conditions during occupation (e.g. was is rainy or dry season), v) prevailing conditions after abandonment—which affects dung decomposition and recolonization dynamics, vi) soil type. All, the factors will affect how utilization patterns of an abandoned boma will unfold. Additionally, only get to learn under the discussion session, that creation of bomas that were used for this study did not involve cutting of trees. This is a novel aspect of this work, since most of abandoned bomas elsewhere are devoid of trees; which affects perceived predation risk. – the setting up of cattle bomas has been explained in details (see L277-288).

Thirdly, it is misleading to state that “few studies have monitored the use of nutrient hotspots created through kraaling cattle in temporary mobile overnight kraals by wildlife”. Indeed, a lot of work has been done on this this subject, e.g see the citations below. Instead, the authors should clarify the novel aspects of their work. – the citations below have been used to correct the misleading statement (see L89-92)

Augustine, David J., Veblen, Kari E., Goheen, Jacob R., Riginos, Corinna, and Young, Truman P. 2011. "Pathways for Positive Cattle–Wildlife Interactions in Semiarid Rangelands." Smithsonian Contributions to Zoology. 55–71. <https://doi.org/10.5479/si.00810282.632.55>

Veblen, K. (2012). Savanna glade hotspots: Plant community development and synergy with large herbivores. Journal of Arid Environments, 78, 119–127.
Donihue, C. M., Porensky, L. M., Foufopoulos, J., Riginos, C., & Pringle, R. M. (2013). Glade cascades: Indirect legacy effects of pastoralism enhance the abundance and spatial structuring of arboreal fauna. Ecology, 94(4), 827–837.

Fourthly, it is not clear how successive photographs of an individual animal or a group of animals were treated. With camera traps, you often get cases of some animals hanging out on front of a camera trap, consequently triggering the camera multiple times. Where these analyzed as independent detections. If so, how about individuals that could possibly have been in the glade, but not immediately in front of a camera trap, hence fewer photographs of them taken. I know this is a thorny issue, but there are number of recommendations about deciding which of the triggers are independent detections. Some studies have set a threshold of 20min, others 30min, and even 1hr. Whatever threshold is used, it is important to be explicit about it – in this study we analysed images of animals captured by the camera trap. We did not use video images, instead we used still pictures. It was therefore not possible to detect animal movements & time in the kraal. However, similar images were treated as one to avoid double counting.


Lastly, in addition to documenting above grass biomass accumulation and compensatory growth, the authors should also have looked at species composition. Growth strategies and response to grazing vary remarkably across different species and we also know that nutrient hotspots like these promote dominance of distinct herbaceous communities, which may actually change over time. – species composition was presented in another manuscript (see Huruba et al. 2018)

Specific suggestions:
Ln 16: delete “natural” – deleted (see L16)
Ln 18 (and multiple other places): “determine” has a connotation of causality inference. Replace with something like “examine) – 'determine' was replaced by 'collated' (see L20)
Ln 27: delete “to ascertain their use of these nutrient hotspots” – deleted (see L21-22)
Ln 35-35: would have been more interesting if this compensatory growth in the hotspots was compared with the matrix – true, unfortunately we don’t have data for the matrix
Ln 35: “Impala benefited the most….”: The fact that they used the hotspots more frequently doesn’t imply the benefited more. Why not just say: “Impala were attracted to …..” – this was corrected (see L27)
Ln 38 (and elsewhere): the conclusion about nutrients hotspots increasing rangeland heterogeneity is not supported by the data presented here. How was rangeland heterogeneity measured? – this has been corrected (see 33-34)
Ln 49: congregate -> are attracted – the sentences was deleted & thus no longer applicable
Ln 53: "lawns, old”: insert coma – the sentences was deleted & thus no longer applicable

Ln 74-74: sentence not clear – the sentence was deleted
Lin 79: there are many studies that report attraction of herbivores to glades. Consider citing some of them here - these have been cited (see L90)
Ln 96-97: not clear what the ‘feedback loop’ is here – this has been explained (see L168-169)
Ln 88-104: sounds repetitive –deleted (see L119-135)
Ln 124-126: contradicts earlier statement? – deleted (see L170-171)
Ln 137: “restricts grass height” -> “maintains grass short” – deleted (see L194)
Ln 174-175: This study was conducted in Debshan ranch, located….. – corrected (see L256-257)
Ln 204-207: hard to understand this sentence –revised (see L299-302)
Ln 207-208: What date was recoded here? –corrected (see L301-302)
Ln 277-278: there is no mention of there this wildlife abundance data is coming from –corrected (see L413-414)
Ln 330: the explanation as to why impala were attracted to 4yr old glades is not quite convincing: what would occasion the changes in grass and browse? –corrected (see L468-470)

**Reviewer #2:** Short duration overnight cattle kraaling in natural rangelands: does time after kraal use affect their utilization by wildlife and above ground grass parameters?


1. The study presents the results of original research

The study investigated the use of previously kraaled sites (aged 1,2,3 and 4 years) by Burchell’s zebra and warthogs (grazers), impala and African savanna elephants (mixed feeders) and northern giraffe and kudu (browsers) during the wet season (January), early dry season (June) and late dry season (October). Camera traps were used to record large herbivore sightings in previously kraaled sites. In addition, an experiment to simulate grazing was carried out to determine the response of grass growth to repeated removal of grass biomass during the 3 stated seasons. The study was carried out at Debshan ranch, a mixed cattle-wildlife ranch in central Zimbabwe. The study presents results of original work.

2. Results reported have not been published elsewhere

To the best of my efforts and checking, I confirm that the results reported in this manuscript have not been published in the peer-reviewed literature. However, it is noted that this manuscript has been deposited in the preprint server bioRxiv. PLOS accepts and supports this practice as per PLOS ONE criteria for publication #2.

3. Experiments, statistics, and other analyses are performed to a high technical standard and are described in sufficient detail.

The study was conducted following the scientific method or process. The data collection methods were described and data analyzed. However, the following needs attention: -

Abstract

Lines 30-32: Please clarify what ‘similarly’ stand for or mean. Results showed that the 8 mammals did not use the previously kraaled sites in the same way. So, what does “similarly; mean? - this has been clarified (see L25-26)

Introduction
• Lines 68-72 This paragraph is too short and is like a hanging paragraph. Please cite more studies that have used camera traps on nutrient hotspot by wildlife. In addition, state some limitations of using camera traps and how you addressed these in your study. Include, if any, how the results of your studies were or were not affected by the use of camera traps – this paragraph has been revised (see L232-240)

• Line 16, 90, 91, while it may be obvious, the use of “Short duration overnight” must be clearly defined or elaborated in the study. Did “Short duration overnight” imply that the cattle were at these spots just for one night hence short duration? If yes, why not refer to these only as “Overnight?”. If cattle were kraaled for few nights at a time then should such number of nights just be stated? This needs to be made clear in the text. – this has been clarified (see L17; L86; L278-288)

• Line 126-27 You refer to previously kraaled sites that are used by grazers that deposit dung and urine as “newly created” nutrient hotspots. Why do you refer to then as newly created? Why not refer to them as “nutrient enriched or recharged previously kraaled sites? I suggest this because authors asserted that kraaling adds nutrients to the soil. So, why does it now become “newly created” when it has been established already by cattle kraaling? – the term 'nutrient enriched hotspots' has been adopted & is used throughout the manuscript (for example, see L18; L86-87; L147; L215 etc). We wish to thank the reviewer for the excellent suggestion.

• Line 153-154. You state that “However, the positive herbivore-grass feedback has not been widely tested, particularly in nutrient hotspots”. If authors had stated.. “has not been tested” If would indicate no testing has been done elsewhere before. However, by stating that it has not been widely tested, it suggests that it has been tested but in a limited way. If this is the case, in what limited way has this positive loop been tested? What were the outcomes and how do these inform or influence your study? – the above statements has been rewritten (see L216-218)


Materials and methods

While the materials and methods are well described in the manuscript, there are some sections that need more detailed attention. These are listed below: -

Line 197 Camera traps setting –

• Line 199-203: Specify how many camera traps were set at each of the previously kraaled sites aged 1,2,3 and 4 years? How many replicates of the previously kraaled sites by age (1,2,3 and 4 years) were established? How many replicates of the control were established and how many camera traps were placed at control sites? – these have been specified (see L 296-298)


Line 219 Estimates of aboveground grass biomass cropped by grazers

• Line 225-226 Specify how much grass was clipped? At what height from the ground was grass cut? Did you cut the grass all the way to the ground or what? Please specify. – this has been specified (see L333-334)

• Line 229. To what nearest measurement was grass height recorded? – this has been specified (see L337)

• Line 231 Specify the height to which grass were clipped inside movable cages at each of the growth season? Or how close to the ground were grass clipped during each growing season? – this has been specified (see L340-341)


243 Statistical analysis

• Line 244: The number of wildlife sightings varied between the 3 sampling seasons i.e January, n=324), June, n=874 and October, n=1635. It is noted that there was huge variation in number sightings such that the October values were 5 times and 2 times larger than the January and June sightings respectively while the June sightings were about 3 times larger than the January values. To what extent did such variation affect the outcomes of the data analysis comparisons? How did this variance affect / influence the outcomes of the data analysis? Did you take into account of this variation and if so how? Please include such in the text. – the numbers presented were those of camera images. This has been explained (see L354-356)

Results

• Line 254-255. You state that Zebra, warthog, impala, elephant, giraffe and grater kudu were the most frequently sighted in the camera traps. The camera must have captured pictures of mammals other than the 6 study animals. Please list the other mammals apart from the 6. The ranch must surely have other mammals that came to the previously kraaled sites. – these have been listed (see L371-373)

• Line 256-258 Authors write….time after kraal use ‘had no significant’ effect…… .In a similar manner and for consistency authors should write …..while wildlife sightings varied significantly with wildlife species…’ [authors should similarly use the word ‘significant’ or ‘not significant’ throughout the results section and discussion where the statistical outcomes reveal so. This will ensure consistency] – this has been done (see L 376; 377; 381; 384; 386; 392

• Line 261. Authors should use early season, early dry season and late dry season instead of months January, June and October. – this has been done (see L378; 382)

• Lines 266-280 Authors refer to statistical outcomes for results plotted in Figure 3 (number of sightings) and Figure 4 (Population).

o Authors must clarify or describe how the sighting was converted to population based on camera trap photos. This has not been described in the methods section. – this has been explained in the methods (see L322-323)
o In line 277, authors must indicate that impala and giraffe were the most and least abundant wildlife species at Debsham ranch as estimated from camera trap pictures in 2016. This is important because Camera trap method has limitations as a method or estimating abundance. – the population was from aerial counts. This has been clarified (see L413-415)
o Authors therefore must state the extent to which population estimates presented in Figure 4 (line 617-618) and accurate based on how many of these mammals are present on the entire Debshan ranch? – this has been clarified (see L413-415)
o Authors need to indicate limitations of such a method to estimate populations of mammals. - aerial census results were used to census the wildlife not camera trap data (see L413-415)

• In the results and discussion authors refer to sighting index including Figure 4 (line 621). Yet this is not explicitly indicated so or referred to as such in the Materials and methods especially under statistical analysis. – this has been included (see L321-323)

• In Figure 2 (line 620), Figure 3 (line 606), Figure 5 (line 621) and Figure 6 (line 631) and Figure 7 (line 637) please indicate / include the word ‘Mean’ on the Y-axis labels. – this has been corrected (see Figures 2, 3, 5, 6 & 7)

Discussion

• Line 302-303 Authors should state the mechanism which Bailey et al [48] suggested to assert that herbivores were able to identify patches with forage of varying nutritive value. How do herbivores identify nutritive value? How can we be sure? This important because it would contribute to understanding choices which mammals in the present study may have used to select and visit nutrient rich hotspots of previously used kraal sites – this statement has been made redundant in the revised manuscript (see L430-433)

• Line 308—310. Consider using the phrase ‘nutrient enriched hotspots” than ‘newly created nutrient hotspots’ when referring to these previously kraaled sites. – this was done (see L429; 431-432; 439; 440-441 etc)

• Line 312-319. You make reference to use of woody plants by giraffes in your study. Why did you not quantify, characterize and compare woody plants at the previously kraaled sites to enable reference to woody plants being preferred and eaten by giraffes? – this could have presented interesting results. However, this study focused on the use of nutrient enriched hotspots only. This could be considered in future studies

• Line 329-335.
o While authors explained preference of impala and elephants for previously used kraal sites that were one year and four years old (only impala) , they did not explain lack of preference of use of sites that were 2 and 3 years old. Please suggest an explanation and discuss. – use of one year old sites could be attributed to their openness, while the other years were not open (see L467-470).
o In line 331 authors state that impala and elephants can switch between grasses and browse depending on their nutritive quality. This in my view is a suggested possibility because the nutritive quality of the forage was not investigated or assessed; it was merely inferred. If this is so, it is inaccurate for authors to then conclude in line 332 that …. Therefore, impala and elephants were able to make choices from previously kraaled sites of varying ages depending on the quality of either grasses or browse. Authors must address this. - this was deleted (see L476-478)

• Line 343-347. Authors should address the issue of estimation of population of study mammals based in camera trap data raised earlier. In these 5 lines, authors have merely stated the results but have not dis used or interpreted the results neither have they made any reference to literature. What do these results mean and what contribution do these add to the use of previously kraaled sites by mammals in this study? – this has been explained (see L544-549)

• Line 352 and 353 In order to assert / suggest the possibility that high aboveground grass biomass may have high risks of predation from ambush-hunting predators such as lions [52] authors must indicate whether lions are present in the Debshan ranch. – this was deleted (see L508-510)
• Line 357-358. Authors must explain why warthogs kept aboveground biomass and grass height low in previously kraaled sites that were 3-4 years old compared to those that were 1 to 2 years old and also in general. Although authors refer to Huruba et al. [3] who reported that warthogs intensely grazed in previously kraaled sites, they did not state why? Elaborate? - this was made redundant by the revisions of the manuscript (see L513-515)

• Line 361 reference Burkepile et al. [53] is given the same reference number of [53] in line 363 for Groom and Harris [53] instead of [54] as indicated in the reference section. Please correct. – this was corrected

4. Conclusions are presented in an appropriate fashion and are supported by the data. -

Conclusions made are generally supported by the data except Line 385 – 386. It is not accurate to conclude that the study has showed that short duration overnight cattle kraaling in natural rangelands creates nutrient hotspots. The use of ‘nutrient’ is inferred and not an outcome of this study because nutrients at the previously kraaled sites were not measured nor quantified and compared but inferred.

- the conclusion was rewritten (see L552-561)

  5. The article is presented in an intelligible fashion and is written in standard English

  This statement is true for this manuscript.


  6. The research meets all applicable standards for the ethics of experimentation and research integrity.

  Ethics of Experimentation
  Authors did not indicate whether they had obtained appropriate research permits and ethical clearance for this study. If they did then they must cite reference numbers for the respective permits and clearance. – permission to conduct the research was granted by the Debshan ranch owners & management. The study was non invasive & thus no unethical practices were used.

  Publication Ethics
  Authors have specified their respective contributions to the study (line 397-407)

  7. The article adheres to appropriate reporting guidelines and community standards for data availability.
  Reporting Guidelines
  Results were rigorously reported, as appropriate based on the type of the data collected.
  Data Availability
  Authors have presented data as expected in a scientific journal. The results of the study did not involve gene sequences etc that are deposited following set standards and practice

6. PLOS authors have the option to publish the peer review history of their article ([what does this mean?](https://journals.plos.org/plosone/s/editorial-and-peer-review-process#loc-peer-review-history)). If published, this will include your full peer review and any attached files.


If you choose “no”, your identity will remain anonymous but your review may still be made public.


**Do you want your identity to be public for this peer review?** For information about this choice, including consent withdrawal, please see our [Privacy Policy](https://www.plos.org/privacy-policy).

Reviewer #1: No

Reviewer #2: No

[NOTE: If reviewer comments were submitted as an attachment file, they will be attached to this email and accessible via the submission site. Please log into your account, locate the manuscript record, and check for the action link "View Attachments". If this link does not appear, there are no attachment files.]

While revising your submission, please upload your figure files to the Preflight Analysis and Conversion Engine (PACE) digital diagnostic tool, <https://pacev2.apexcovantage.com/>. PACE helps ensure that figures meet PLOS requirements. To use PACE, you must first register as a user. Registration is free. Then, login and navigate to the UPLOAD tab, where you will find detailed instructions on how to use the tool. If you encounter any issues or have any questions when using PACE, please email PLOS at [figures@plos.org](mailto:figures@plos.org). Please note that Supporting Information files do not need this step.
