# Supplementary material for: Short duration overnight cattle kraaling in natural rangelands: Does time after kraal use affect their utilization by wildlife and above ground grass parameters?
Source: PLoS One. 2022 Apr 28;17(4):e0248795. doi: 10.1371/journal.pone.0248795 (PMC9049567; doi:10.1371/journal.pone.0248795)
Supplement: Attachment — Submitted filename: response to reviewer comments.doc [file pone.0248795.s002.doc]

**Response to reviewer comments- PONE-D-21-06800R1**

**Short duration overnight cattle kraaling in natural rangelands: does time after kraal use affect their utilization by wildlife and above ground grass parameters?**

Reviewer #1:

*The authors have done fairly good job in incorporating suggestions from earlier reviews, there still remains substantial challenges in how the data was analyzed and how the results are presented. – the data analysis & results presentation has been corrected* (see data analysis & results L249-264; L270-271; L274-305)

*First, I still don’t think the concern about how camera trap data was analyzed was adequately addressed. In their response latter, the authors state that “similar images were treated as one to avoid double counting” but there is no mention of this in the manuscript. Again, what does “similar” mean? If a camera trap captures two photographs within a period of 60 seconds, in one photograph there are three impalas and in the subsequent photograph there are five impalas; are there ‘similar images’? Essentially, multiple detections during a single short occasion are not likely to be independent and thus may contribute little information or may bias estimates. Most studies set a minimum time threshold for independent detections; e.g. all successive photographs of one individual (or species) taken within 10 mins are treated as a simple detection (photographic event). There is plenty of literature around this topic and I encourage authors to look at look at this* – this has been corrected (see L200-201)

*Inferences are made about differences across herbivore guilds, in utilization of kralls. However, there are no results to support this, and the authors do not specify which of the six herbivores belongs to which guild.* – the herbivores in the three feeding guilds have been stated (see L136-137)

*Ln 243-244: “general linear model (GLM) univariate” -> “univariate general linear model (GLM)”: again, why use univariate models? A more appropriate approach would have been to fist a model that includes all the variables and interactions* – the GLM univariate analysis & results were removed while only remaining with one way analysis of variance (see L249-251).

*Ln 244 “effects” -> “differences”?* – this was corrected (see L250)

*Ln 247: I did not see any post hoc analysis presented anywhere in the results* – the post hoc results are now shown (see Figure 2 & 3 L596-597; L604-605)

*247-249: why just zebra and warthog?* – these were the only grazers (see L261)

*260-261: I don’t see much value in testing the differences in detections across different wildlife species. However, if the authors choose to retain this, they should at least include multiple comparison tests and state which species were actually different* – the comparison across different wildlife species were removed (see L274-276).

*Ln 262-263: rephrase for clarity e.g “For each of the six species, there were no significant differences in sightings across the three seasons [early (January), early dry (June) and late dry (October)]…..”* – this has been corrected see (L277-282).

*Ln 265-267: this should be presented above ln 260-261* – this has been changed (see L282-284).

*The authors need to resolve inconsistent use of scientific and common names in reference to different animals e.g. Ln 257-258, ln 272-273 (and elsewhere): The common practice is to give the two names the first time a species is mentioned, and subsequently refer to on one* – this has been corrected (see L154-157)

*Line: 274-279: it is not clear what is being tested here. Rephrase for clarity* – the results have been rewritten to improve on clarity (see L268-305).

*Ln 280-284: I find these two statements, just like the most of the other statements under results section, quite confusing.* – the results have been rewritten to improve on clarity (see L268-305)

*Ln 285-285: how is this “evidence that most grazing occurred in the three-year-old nutrient enriched hotspots.”?* – this sentence was deleted (see L302-303).

*In 282-283, the authors state that three-year-old kralls has the most biomass (most productive), while in 286-287, they state that one year old Kralls had the tallest grass. How is this possible? I thought biomass was correlated with grass height?* – this has been corrected (see L302-303)

*Ln 287: new paragraph?* – this was corrected
*292-293: I still don’t see what value the value of comparing camera trap and aerial census data* – Aerial census data was used to calculate sighting indices & was not compared with camera trap data (see L393-398).

294-295: not clear – this has been corrected
